# Development and validation of the AI-assisted psychoemotional regulation scale (ERP-IA-6)

**DOI:** 10.3389/fpsyg.2026.1917798

**Published:** 2026-09-09

**Authors:** Berle Estalin Briones-Llamoctanta, Jonathan Andrés Pacheco-Cavero, Victor Saul Callacondo-Quispe, Josué Edison Turpo-Chaparro

**Affiliations:** 1 Professional School of Psychology, Universidad Peruana Unión, Lima, Peru; 2 Universidad Peruana Unión, Lima, Peru; 3 Universidad Peruana Unión, Juliaca, Peru; 4 Faculty of Human Sciences and Education, Universidad Peruana Unión, Lima, Peru

**Keywords:** adults, artificial intelligence, emotion regulation, psychometrics, reliability, scale development

## Abstract

**Background:**

Conversational and generative artificial intelligence tools are increasingly used to organize thoughts, clarify emotional experiences, and cope with everyday distress. However, brief instruments assessing perceived AI-assisted psychoemotional regulation remain scarce.

**Objective:**

To develop the AI-Assisted Psychoemotional Regulation Scale (ERP-IA-6) and examine its initial psychometric properties among Peruvian adults.

**Methods:**

The initial eight-item pool was reviewed by five psychologists, and all items obtained Aiken’s V values above 0.80. A total of 538 adults participated (M age = 24.51 years, SD = 7.48; range = 18–81); 81.6% were aged 18–29 years, 52.0% were women, and 93.1% reported AI use. Using a reproducible sex-stratified procedure, the sample was divided into exploratory (*n* = 266) and confirmatory (*n* = 272) subsamples. Exploratory factor analysis used polychoric correlations and unweighted least squares; confirmatory factor analysis used WLSMV for ordinal indicators. Reliability, AVE-based convergent evidence, sensitivity analyses, shared-method diagnostics, and ordinal measurement invariance across sex were also examined.

**Results:**

Exploratory analyses supported reducing the scale from eight to six items based on conceptual redundancy and parsimony. The final version showed adequate factorability (KMO = 0.910; Bartlett’s χ^2^ (15) = 1128.37, *p* < 0.001) and a one-factor solution explaining 67.4% of standardized item variance, with loadings from 0.750 to 0.872. In the confirmatory subsample, loadings ranged from 0.839 to 0.930. Robust incremental and residual fit were favorable (CFI = 0.981; TLI = 0.968; SRMR = 0.019), although robust RMSEA was elevated, 0.118, 90% CI [0.093, 0.145]. Sensitivity analyses showed that the one-factor structure persisted, while localized residual dependence partly accounted for the elevated RMSEA. Reliability was high (α = 0.935; ordinal α = 0.945; ω = 0.948), as were composite reliability (CR = 0.959) and average variance extracted (AVE = 0.794). Threshold, metric, and scalar models provided favorable but cautious evidence of invariance across sex.

**Conclusion:**

The ERP-IA-6 provides favorable initial evidence as a brief measure with a dominant unidimensional structure for assessing perceived AI-assisted psychoemotional regulation among Peruvian adults. Scores should be interpreted as perceived regulatory support, not as evidence of clinical effectiveness, therapeutic safety, or equivalence to professional care.

## 1 Introduction

The rapid expansion of conversational artificial intelligence (AI) has transformed the ways in which people seek information, organize their thoughts, express distress, and cope with everyday emotional difficulties (Boyd and Markowitz, 2026; Santiago-Torner, 2026). Although younger people and university students have been especially prominent in the available literature because of their high digital familiarity and the convergence of academic, economic, and psychosocial stressors that may increase emotional vulnerability, AI-mediated emotional interaction is not conceptually restricted to young adulthood (Türk et al., 2025; Yeung et al., 2025). In this context, AI-based conversational agents have begun to occupy an increasingly visible yet ambiguous role: on the one hand, they function as accessible, immediate, and low-threshold tools for emotional support; on the other hand, they remain insufficiently understood in terms of their psychological, relational, and self-regulatory effects (Volpato et al., 2025). Recent literature suggests that the field should no longer focus only on whether AI “works” in clinical terms, but also on how these technologies become integrated into users’ emotion regulation processes and everyday psychological experience, particularly in conversational interaction contexts (Jenko et al., 2025; Maleki Varnosfaderani and Forouzanfar, 2024).

Current developments in affective artificial intelligence are shifting digital systems from merely conversational functions toward architectures capable of recognizing, modeling, and responding to emotional states through multimodal integration, personalized profiles, and controlled generation of socioemotional content (Zhao et al., 2025). This transition has begun to show applied implications in educational and mental health contexts. AI-supported environments, including ChatGPT and Bing, have been associated with improvements in interpersonal emotion regulation, behavioral emotion regulation strategies, reduced hopelessness, and vocabulary retention among students of English as a foreign language (Wang and Akhter, 2025). Similarly, a cognitive behavioral therapy-based chatbot reduced depression and loneliness among Chinese university students, particularly among those experiencing higher financial stress (Wang et al., 2025). Nevertheless, available reviews caution that these effects should be interpreted carefully, given the persistence of methodological heterogeneity, high attrition, reliance on self-report measures, and the limited proportion of systems with rigorous clinical validation, especially among large language model-based tools (Hua et al., 2025; Nyakhar and Wang, 2025).

Beyond clinical effectiveness, a central issue for human–AI interaction research is that AI is reshaping the emotional ecologies of everyday life. Santiago-Torner (2026) argues that, between 2020 and 2025, AI altered access to emotional support, affective expression, and the construction of interpersonal trust, generating a sociotechnical ecology in which opportunities for personalization and accessibility coexist with risks of pseudo-empathy, affective dependence, and erosion of relational authenticity. This perspective converges with user-centered design approaches, such as that of Vafafar (2025), who showed that personalized emotional assistance through chatbots cannot rely on generic prompts alone, but requires contextual sensitivity, cultural adaptation, transparency, and alignment with users’ lived experience. In other words, AI-mediated emotional support should not be understood merely as a form of “digital intervention,” but as an emerging form of human–technology interaction in which processes of reflection, reappraisal, and emotional coping may be externally scaffolded (Boyd and Markowitz, 2026; Martínez et al., 2026).

The construct of AI-assisted psychoemotional regulation can be grounded in the process model of emotion regulation and, more specifically, in its extended formulation, according to which regulating an emotion involves influencing which emotion emerges, when it occurs, and how it is experienced or expressed through successive phases of identification, selection, implementation, and monitoring of the regulatory process (Gross, 2002; Rodriguez and Kross, 2023). This architecture is particularly relevant because regulatory difficulties do not appear as isolated failures, but rather as sequential disruptions that may be located within these phases and may propagate from early identification deficits to later problems in the selection and implementation of strategies (Agathos et al., 2025; Cai and Samson, 2025). Moreover, emotion regulation has been recognized as a relevant transdiagnostic mechanism in cognitive behavioral interventions for anxiety and depression, reinforcing its explanatory value beyond specific clinical categories (Reinholt et al., 2025). From this perspective, AI is not conceptualized as an emotion, an autonomous affective agent, or a therapeutic substitute, but rather as an external scaffold that may assist specific regulatory tasks, such as naming what one feels, organizing thoughts, reinterpreting a situation, or modulating impulsive responses. This interpretation is consistent both with models of intelligent agents designed to facilitate extrinsic emotion regulation within Gross’s framework (Pico et al., 2024) and with the emerging development of automated emotion regulation systems based on multimodal sensors and large language models, although this field still faces substantial ethical, technical, and practical challenges (Yu et al., 2025).

The importance of studying this construct increases when considering that emotion regulation has been recognized as both a target and a mechanism of change in multiple interventions. Reinholt et al. (2025) found that specific emotion regulation skills predict symptomatic improvement in group therapies for anxiety and depression. Likewise, emerging reviews suggest that AI-assisted interventions may contribute to improving emotion regulation, reducing rumination, and promoting emotional self-control among students, although the available evidence remains preliminary and methodologically heterogeneous (Fakouri et al., 2026). Along the same lines, a recent meta-analysis on AI conversational agents and stress reduction concluded that the effects are promising but contextually variable, with mixed results depending on population, design, and intensity of use (Von Ahlefeldt et al., 2026). Taken together, these findings indicate that emotion regulation is not a peripheral variable in AI interaction, but rather a plausible pathway through which these tools may produce subjective and behavioral effects.

However, a psychometric problem remains. The most established instruments in the field continue to assess general emotion regulation strategies rather than the specific mediation of AI. The Process Model of Emotion Regulation Questionnaire (PMERQ) was developed to measure individual differences in the use of strategies distributed across different stages of the process model, and its German adaptation showed a predominantly adequate structure and sufficient reliability, confirming the relevance of fine-grained measurement of the regulatory process (Heekerens et al., 2024; Olderbak et al., 2023). Similarly, the Emotion Regulation Questionnaire-8 (ERQ-8) has shown satisfactory psychometric properties among Chinese university students, although its cross-national invariance is not uniformly supported, underscoring the need for contextual validation before transferring regulatory constructs to new settings (Burghart et al., 2023; Zhao et al., 2024). These measures assess general regulatory strategies independently of the technological resource through which regulation may be supported. Merely replacing their original referents with AI-related wording would alter the meaning of the items and scores without demonstrating that the resulting content adequately represents AI-assisted regulation. Scale-development recommendations emphasize that item content must follow a clearly delimited construct definition and remain distinguishable from adjacent constructs rather than being adapted only through superficial wording changes (Clark and Watson, 2019; Flake and Fried, 2020). Thus, the literature provides instruments to measure how people regulate their emotions in general, but it still lacks a brief and specific measure of the extent to which AI is perceived as supporting that process.

The need for a new instrument becomes even more evident when reviewing emerging scales on human–AI interaction. Recent measures have focused mainly on dependence, attachment, or problematic use. The Conversational AI Dependence Scale (CAIDS) was designed to assess dependence on conversational AI (Y. Chen et al., 2025); the Problematic ChatGPT Use Scale focuses on maladaptive patterns of use (Maral et al., 2025); the Generative AI Dependency Scale examines dependence through motivational, behavioral, and psychological correlates (Goh et al., 2025); the AI Attachment Scale evaluates emotional closeness, social substitution, and normative consideration toward AI (Kasturiratna and Hartanto, 2026); and the Emotional Trust in Artificial Intelligence Scale (CEIA) evaluates emotional dependence on AI (Briones-Llamoctanta et al., 2026). Although these instruments are valuable, their intended score interpretations concern problematic involvement, psychological reliance, emotional attachment, or relational substitution. They were not designed to assess the functional use of AI as a cognitive and emotional scaffold for identifying, clarifying, reinterpreting, and modulating emotional states. Consequently, adapting these measures would risk conflating regulatory support with dependence or attachment and would not preserve their original construct meaning. Therefore, there is a specific conceptual and measurement gap between assessing a potentially problematic relationship with AI and assessing its perceived instrumental incorporation into psychoemotional self-regulation.

This gap is critical because emotional support mediated by generative AI is not merely a neutral extension of digital support, but an emerging form of interaction capable of activating trust, pseudo-empathy, and socioemotional projection. These processes may intensify the subjective perception of support, but they may also foster artificial validation, affective dependence, or displacement of interpersonal judgment (Saracini et al., 2025; Volpato et al., 2025). From a psychotherapeutic perspective, although AI may simulate components of listening, validation, and mentalization, structural limits remain regarding intentional reciprocity, affective presence, and moral commitment. This makes it necessary to empirically distinguish between regulatory use, attachment, dependence, and relational substitution (Yirmiya and Fonagy, 2025). Consequently, measuring AI-assisted psychoemotional regulation represents a theoretical, psychometric, and ethical necessity, as it allows researchers to examine whether these tools are perceived as scaffolds for recognizing, organizing, understanding, and modulating everyday emotional experiences, rather than assuming that any emotionally oriented interaction with AI necessarily reflects dependence or relational compensation.

Accordingly, the present study aimed to develop the AI-Assisted Psychoemotional Regulation Scale and examine its initial psychometric properties among Peruvian adults. The instrument was developed as a brief measure of perceived AI-assisted psychoemotional regulatory support, while its dimensional structure was evaluated empirically. Specifically, the study examined content-validity evidence, item performance, internal structure through exploratory and confirmatory factor analyses conducted in independent subsamples, score reliability, convergent validity, and measurement invariance across sex. In doing so, the study seeks to contribute to the literature on human–AI interaction from a psychological and psychometric perspective by differentiating AI-assisted regulatory support from classical emotion-regulation strategies and from emerging measures of attachment, dependence, and problematic AI use. Given the convenience sampling procedure, the concentration of participants in younger age ranges, and the absence of external criterion measures, the findings are interpreted as initial evidence requiring replication in more diverse adult populations.

## 2 Method

### 2.1 Study design

An instrumental, cross-sectional psychometric study was conducted to develop and examine the initial measurement properties of the AI-Assisted Psychoemotional Regulation Scale (ERP-IA). Instrumental studies are appropriate for the construction, refinement, and evaluation of psychological measures through multiple sources of validity and reliability evidence (Ato et al., 2013).

The development process comprised conceptual definition, initial item generation, expert content review, empirical administration, data screening, exploratory item refinement, confirmation of the retained structure in an independent subsample, reliability assessment, sensitivity analyses, and measurement-invariance testing. The separation of exploratory and confirmatory procedures was intended to reduce capitalization on sample-specific characteristics (Boateng et al., 2018; Worthington and Whittaker, 2006).

### 2.2 Participants

The analytic sample comprised 538 Peruvian adults aged 18 years or older who voluntarily provided digital informed consent and completed the study questionnaire. The mean age was 24.51 years (SD = 7.48), the median was 22 years, the interquartile range was 20–26 years, and the observed range was 18–81 years. Because eligibility was defined by adulthood rather than a restricted developmental period, the sample is described throughout the revised manuscript as Peruvian adults, although it was predominantly concentrated in younger age ranges.

The sample included 280 women (52.0%) and 258 men (48.0%). Regarding educational attainment, 432 participants reported university education (80.3%), 46 technical education (8.6%), 40 secondary education (7.4%), and 20 postgraduate education (3.7%). A total of 501 participants (93.1%) reported using AI tools, whereas 37 (6.9%) reported no AI use. Frequency of use was low for 37 participants (6.9%), moderate for 372 (69.1%), and high for 129 (24.0%).

Participants were recruited through nonprobability convenience sampling, a procedure based on the accessibility and voluntary availability of eligible participants (Etikan et al., 2015). Inclusion criteria were: (a) being 18 years of age or older; (b) providing informed consent; and (c) completing the ERP-IA items. Records were designated for exclusion if they lacked affirmative consent, belonged to a participant younger than 18 years, contained missing or invalid ERP-IA responses, included values outside the 1–5 response range, had repeated identifiers, or constituted exact duplicate records.

The data-screening process identified no records meeting these exclusion criteria; therefore, all 538 participants were retained. Coincident item-response profiles among records with different identifiers were not considered duplicates because response similarity alone did not demonstrate invalid participation.

The total sample was considered sufficient for the planned analyses given the small number of indicators and simulation evidence showing that factor-analytic sample requirements depend on model complexity, factor loadings, communalities, and estimation conditions rather than on a universal participant-to-item ratio (MacCallum et al., 1999; Wolf et al., 2013). Both the exploratory and confirmatory subsamples contained more than 250 participants.

### 2.3 Scale development and content-validity evidence

#### 2.3.1 Construct definition and initial item generation

AI-assisted psychoemotional regulation was defined as the extent to which individuals perceive that AI tools help them identify, organize, understand, reinterpret, or modulate everyday emotional experiences. The construct was informed by process-oriented models in which emotion regulation involves the identification of emotional experiences, selection and implementation of regulatory responses, and monitoring of their effects (Gross, 2002; Rodriguez and Kross, 2023).

The research team initially generated eight positively worded items after reviewing the theoretical model and emerging literature on conversational and generative AI as resources for emotional clarification, cognitive organization, and everyday self-regulation. Identification, selection, implementation, and monitoring were used as an initial theoretical content map. However, these processes were not scored as established subscales, and the dimensional structure of the item pool was treated as an empirical question.

Items were written in brief and accessible language for adult users without requiring specialized knowledge of psychology or AI, following general recommendations for the development of clear and construct-relevant scale items (DeVellis, 2017). Content referring directly to emotional dependence, compulsive use, interpersonal replacement, or affective attachment to AI was avoided because these constructs were considered related but conceptually distinct from perceived regulatory support.

#### 2.3.2 Expert review and content validity

The initial eight-item pool was independently reviewed by five psychologists with expertise relevant to psychological assessment and the intended construct. The experts evaluated item wording, relevance, and representativeness. Agreement was quantified using Aiken’s V, with higher coefficients indicating greater agreement regarding item adequacy (Aiken, 1985). All eight items obtained Aiken’s V coefficients greater than 0.80 and were retained for empirical administration. Expert comments were also used to clarify wording and reduce ambiguity.

#### 2.3.3 Response format and scoring

The initial ERP-IA comprised eight items rated on a five-point Likert-type scale ranging from 1 (strongly disagree) to 5 (strongly agree). Following the exploratory analyses, two items were removed because of conceptual overlap with functions already represented by the retained indicators. The final ERP-IA-6 comprises six items.

A total score may be obtained by summing or averaging the six responses, with higher scores indicating greater perceived use of AI as support for psychoemotional regulation. The score does not represent clinical effectiveness, therapeutic benefit, emotional dependence, or the objective quality or safety of AI-provided support.

### 2.4 Procedure

Data were collected through an online self-administered questionnaire. Before entering the survey, participants received information about the study purpose, voluntary participation, confidentiality, academic use of the data, minimal anticipated risk, and the right to discontinue without penalty. Only participants who provided digital informed consent were allowed to continue.

The questionnaire included sociodemographic variables, questions about AI use and frequency of use, and the eight initial ERP-IA items. Responses to the scale items were required by the form to minimize item-level missingness, although participants could discontinue participation at any time.

Before analysis, consent responses and participant age were verified. ERP-IA responses were examined for missingness, numeric conversion, integer coding, and values outside the permitted range. Identifiers were checked for repetition, and exact duplicates were evaluated across all available variables.

Uniform response patterns were identified by counting the number of distinct response categories used across the items. These patterns were not automatically classified as invalid because uniform endorsement may represent a legitimate response to a brief and homogeneous instrument. Their potential influence was instead examined through sensitivity analyses.

### 2.5 Ethical considerations

The study followed the ethical principles for research involving human participants established in the Declaration of Helsinki, including voluntary informed consent, protection of privacy and confidentiality, and minimization of potential risk (World Medical Association, 2024).

Participation was anonymous and voluntary, and no direct personal identifiers were collected. Data were used exclusively for academic purposes and analyzed in aggregate form. Because the study involved self-reported perceptions without an experimental intervention, the anticipated risk was considered minimal. Participation was restricted to adults aged 18 years or older.

The research protocol was approved by the Ethics Committee of Universidad Peruana Unión under approval number 2026-CE-FACIHED-00001.

### 2.6 Data analysis

#### 2.6.1 Software and reproducibility

Analyses were conducted in R version 4.5.3 (R Core Team, 2026). Data processing and exportation were performed using the readxl, dplyr, tidyr, and openxlsx packages. The psych package was used for descriptive analyses, polychoric correlations, parallel analysis, exploratory factor analysis, reliability estimates, and response-pattern diagnostics (Revelle, 2026). The lavaan package, version 0.6–21, was used for confirmatory factor analysis, sensitivity analyses, and measurement invariance (Rosseel, 2012). Data management procedures involving dplyr were supported by Wickham et al. (2014).

The complete R script, fixed random seed, package versions, session information, analytic decision sequence, codebook, and supplementary statistical outputs are provided as reproducibility materials.

#### 2.6.2 Preliminary item analysis

Item analysis included the mean, standard deviation, median, observed range, skewness, kurtosis, and response-category frequencies. Floor and ceiling effects, corrected item–total correlations, Cronbach’s alpha if an item was deleted, and the average interitem correlation were also estimated.

Because the items used five ordered response categories, factor analyses were based on polychoric correlations, which are more appropriate than Pearson correlations for estimating associations between ordinal indicators (Flora and Curran, 2004).

#### 2.6.3 Cross-validation strategy

The sample was divided into two nonoverlapping subsamples using a reproducible sex-stratified random procedure with the fixed seed “20,260,803.” The exploratory subsample comprised 266 participants, including 138 women and 128 men. The confirmatory subsample comprised 272 participants, including 142 women and 130 men.

The initial eight-item pool was examined and refined exclusively in the exploratory subsample. The retained six-item structure was subsequently evaluated without further item deletion in the independent confirmatory subsample.

#### 2.6.4 Exploratory factor analysis and item reduction

Factorability was evaluated using the Kaiser–Meyer–Olkin index, item-level measures of sampling adequacy, and Bartlett’s test of sphericity. The number of factors was determined primarily through parallel analysis and theoretical interpretability. Parallel analysis compares observed eigenvalues with those generated from random data and retains factors whose observed eigenvalues exceed their random counterparts (Horn, 1965).

Exploratory factor analysis was estimated using unweighted least squares on the polychoric correlation matrix. The initial eight-item solution was evaluated before examining the final six-item version. Item reduction was based on the convergence of qualitative and statistical evidence, including conceptual overlap, high interitem associations, preservation of theoretical content, factor loadings, communalities, residual correlations, explained variance, and changes in model fit.

Items re7 and re8 were removed because they duplicated regulatory content already represented by the retained indicators. Their exclusion was not based on low factor loadings but on conceptual parsimony supported by the exploratory comparisons. No item was removed after the CFA.

The exploratory solutions were evaluated using factor loadings, communalities, explained variance, RMSR, residual correlations, TLI, and RMSEA with its 90% confidence interval. No single index was used as an isolated criterion for retaining or rejecting the model.

#### 2.6.5 Confirmatory factor analysis

The final one-factor, six-item model was evaluated in the independent confirmatory subsample. Items were declared as ordered categorical indicators and estimated using weighted least squares with mean- and variance-adjusted statistics (WLSMV), theta parameterization, and a standardized latent-factor variance.

WLSMV was selected because it models ordinal response categories without treating them as continuous and normally distributed. In `lavaan`, the procedure applies diagonally weighted least squares for parameter estimation with robust standard errors and a mean- and variance-adjusted model test. Robust weighted least-squares estimation is recommended for CFA models involving ordinal indicators with a limited number of categories (Flora and Curran, 2004).

Model fit was evaluated using robust (\chi^2), CFI, TLI, RMSEA with its 90% confidence interval, and SRMR. Values near or above 0.95 for CFI and TLI and below 0.08 for SRMR were treated as general reference points. RMSEA was interpreted jointly with model degrees of freedom, estimator, residual diagnostics, and the remaining indices because it can perform unfavorably in models with few degrees of freedom and may also vary across estimation methods (Kenny et al., 2015; Shi and Maydeu-Olivares, 2020).

Standardized factor loadings and item-level (R^2) values were examined. Modification indices were used only to identify possible localized dependence and were not treated as sufficient justification for modifying the primary model.

#### 2.6.6 Sensitivity analyses

Three CFA sensitivity analyses were conducted. First, the model was re-estimated after temporarily excluding participants who selected the same response category across all six items. Second, ULSMV was used to evaluate whether the fit pattern depended on estimator choice. Third, a diagnostic model freely estimated the residual covariance between re1 and re2, the only residual association with a modification index greater than 10.

The correlated-residual model was considered diagnostic rather than confirmatory because the parameter was identified *post hoc* and required substantive justification based on item content.

#### 2.6.7 Reliability and convergent validity

Reliability was evaluated using Cronbach’s alpha, standardized alpha, ordinal alpha based on polychoric correlations, model-based omega, corrected item–total correlations, alpha if an item was deleted, and the average interitem correlation (McDonald, 2013).

Composite reliability and average variance extracted were calculated from the unrounded standardized loadings and residual variances of the confirmatory model. Values of at least 0.70 for omega and composite reliability and at least 0.50 for average variance extracted were considered favorable reference points (Fornell and Larcker, 1981).

#### 2.6.8 Measurement invariance across sex

Measurement invariance was examined across women and men in the confirmatory subsample. Sex was selected because the groups were sufficiently balanced and because establishing score equivalence is necessary before interpreting potential sex-based differences in emotion-regulation and technology-mediated coping.

All response categories were represented for every item in both groups. Four sequential ordinal models were estimated: configural invariance; threshold invariance, constraining item thresholds; metric invariance, constraining thresholds and factor loadings; and scalar invariance, additionally constraining item intercepts.

Models were estimated using WLSMV with theta parameterization. Robust (\chi^2), CFI, TLI, RMSEA, SRMR, and robust scaled chi-square difference tests were reported. Invariance decisions were based on the joint examination of nested-model tests and changes in fit indices. A decrease in CFI greater than 0.010 or an increase in RMSEA greater than 0.015 was considered evidence of possible noninvariance, together with meaningful changes in SRMR (Chen, 2007).

Age-based invariance was not examined because the sample was predominantly concentrated among younger adults and did not provide sufficiently balanced groups across later stages of adulthood.

#### 2.6.9 Shared-method and response-pattern diagnostics

Because all ERP-IA items were positively worded self-report indicators administered in the same questionnaire, shared-method influence was examined descriptively. A single-factor analysis of the total-sample polychoric matrix was used to quantify the variance accounted for by the dominant factor.

Although the initial theoretical framework included distinguishable regulatory processes, the empirical analyses supported a dominant single-factor structure. Therefore, the proportion of variance explained by the first factor could not distinguish substantive integration from shared-method variance and was not interpreted as a definitive test of common-method bias.

Uniform response patterns, average interitem correlations, reliability estimates, factor loadings, and sensitivity-model results were examined jointly to determine whether the observed structure could be attributed exclusively to response-style effects.

## 3 Results

### 3.1 Participant characteristics

The analytic sample comprised 538 Peruvian adults who provided informed consent and completed all study variables. Most participants were aged 18–29 years (*n* = 439, 81.6%), whereas 99 participants (18.4%) were aged 30–81 years. Thus, the sample was predominantly composed of younger adults but was not restricted to a young-adult age group. The sample included 280 women (52.0%) and 258 men (48.0%). Regarding educational attainment, 432 participants reported university education (80.3%), 46 technical education (8.6%), 40 secondary education (7.4%), and 20 postgraduate education (3.7%). A total of 501 participants (93.1%) reported using artificial intelligence tools, whereas 37 (6.9%) reported no AI use. Frequency of AI use was predominantly moderate (69.1%), followed by high (24.0%) and low use (6.9%) (Table 1).

**Table 1:** Participant characteristics of the total analytic sample.

Characteristic	*n*	%
Total sample	538	100.0
Age, years		
18–29	439	81.6
30–81	99	18.4
Sex		
Women	280	52.0
Men	258	48.0
Educational level		
Secondary education	40	7.4
Technical education	46	8.6
University education	432	80.3
Postgraduate education	20	3.7
Reported AI use		
Yes	501	93.1
No	37	6.9
Frequency of AI use		
Low	37	6.9
Moderate	372	69.1
High	129	24.0

Age: M = 24.51, SD = 7.48, median = 22, interquartile range = 20–26, range = 18–81 years. The frequency-of-use item was required by the online form and was therefore completed by all participants, including those who reported no AI use. AI-use variables were reported descriptively and were not included in the psychometric models. Percentages may not total exactly 100 because of rounding.

Because the frequency-of-use item was required by the online form, it was completed by all participants, including those who reported no AI use. AI-use status and frequency were therefore reported descriptively and were not included as eligibility criteria or variables in the psychometric models.

The data-screening procedure identified no missing responses, invalid item scores, participants younger than 18 years, repeated identifiers, or exact duplicate records. Consequently, all 538 records were retained for the primary analyses.

### 3.2 Preliminary item analysis

All six retained ERP-IA items used the complete five-category response range. Item means ranged from 2.90 to 2.99, standard deviations ranged from 1.03 to 1.10, and the median response was 3 for each item. Corrected item–total correlations ranged from 0.780 to 0.843, indicating strong item discrimination. Cronbach’s alpha if an item was deleted ranged from 0.918 to 0.926; therefore, removal of any individual item did not increase the internal consistency of the six-item scale. The average interitem correlation was 0.705 (Table 2).

**Table 2:** Descriptive statistics, item discrimination, and factor loadings of the ERP-IA-6.

Item	*M*	*SD*	Corrected item–total *r*	α if deleted	MSA	EFA loading	*h* ^2^	CFA loading	*R* ^2^
re1	2.95	1.06	0.798	0.924	0.900	0.872	0.761	0.839	0.704
re2	2.90	1.04	0.808	0.922	0.917	0.810	0.657	0.889	0.791
re3	2.94	1.03	0.796	0.924	0.911	0.802	0.644	0.893	0.797
re4	2.98	1.03	0.843	0.918	0.905	0.863	0.744	0.930	0.866
re5	2.99	1.05	0.780	0.926	0.928	0.750	0.562	0.897	0.805
re6	2.91	1.10	0.816	0.922	0.907	0.823	0.677	0.896	0.802

*N* = 538 for descriptive statistics and item discrimination; *n* = 266 for EFA; *n* = 272 for CFA. MSA = measure of sampling adequacy; EFA = exploratory factor analysis; CFA = confirmatory factor analysis; h^2^ = communality. All CFA loadings were statistically significant, *p* < 0.001.

### 3.3 Item reduction and exploratory factor structure

The sample was divided using a reproducible sex-stratified random procedure into an exploratory subsample (*n* = 266; 138 women and 128 men) and an independent confirmatory subsample (*n* = 272; 142 women and 130 men). Item reduction was conducted exclusively in the exploratory subsample, and no further items were deleted after examination of the confirmatory results.

The initial eight-item pool was first evaluated using parallel analysis and exploratory factor analysis based on polychoric correlations. Parallel analysis supported one factor, with a first observed eigenvalue of 5.78 and a second eigenvalue of 0.28. In the one-factor eight-item solution, standardized loadings ranged from 0.773 to 0.894, communalities ranged from 0.598 to 0.799, and the factor explained 69.0% of the standardized item variance. Model residuals were small (RMSR = 0.028), although the TLI (0.932) and RMSEA (0.128, 90% CI [0.105, 0.153]) indicated some remaining model misfit.

The strongest polychoric associations were observed among items re6, re7, and re8, with correlations ranging from 0.754 to 0.818. Items re7 and re8 were judged to overlap conceptually with content already represented by the retained items. Their removal was therefore based primarily on conceptual redundancy and the intended brevity of the measure, with the statistical analyses used to verify that the reduction did not substantially weaken the factor solution. The six-item version retained 67.4% of the standardized variance, compared with 69.0% for the eight-item version, while showing improved exploratory fit (RMSR = 0.020 vs. 0.028; TLI = 0.969 vs. 0.932; RMSEA = 0.093 vs. 0.128).

**Figure 1:**
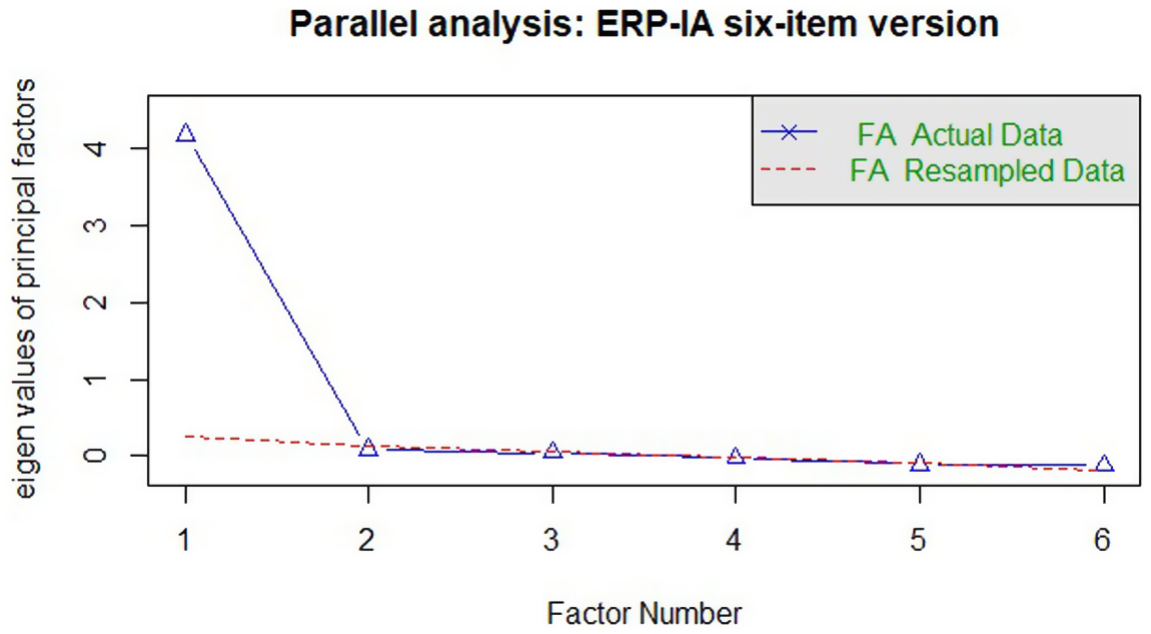
Parallel analysis.

For the final six-item version, the Kaiser–Meyer–Olkin index was 0.910, with item-level measures of sampling adequacy ranging from 0.900 to 0.928. Bartlett’s test of sphericity was significant, χ^2^(15) = 1128.37, *p* < 0.001. Parallel analysis again retained one factor; the first observed eigenvalue was 4.19, whereas the second was 0.09 (Figure 1).

A one-factor model was subsequently estimated using unweighted least squares on the polychoric correlation matrix. Factor loadings ranged from 0.750 to 0.872, communalities ranged from 0.562 to 0.761, and the factor explained 67.4% of the standardized item variance. The residual-based fit was favorable (RMSR = 0.020), and no residual correlation exceeded |0.10|. The largest absolute residual was 0.055. The TLI was 0.969, whereas the RMSEA was 0.093, 90% CI [0.057, 0.131] (Table 3).

**Table 3:** Internal-structure evidence for the initial and final ERP-IA versions.

Model	*n*	Loading range	Variance explained	RMSR/SRMR	TLI	CFI	RMSEA [90% CI]
Initial eight-item EFA, ULS	266	0.773–0.894	69.0%	0.028	0.932	—	0.128 [0.105, 0.153]
Final six-item EFA, ULS	266	0.750–0.872	67.4%	0.020	0.969	—	0.093 [0.057, 0.131]
Final six-item CFA, WLSMV	272	0.839–0.930	—	0.019	0.968	0.981	0.118 [0.093, 0.145]

The CFA robust model test was χ^2^(9) = 72.03, *p* < 0.001. EFA was based on polychoric correlations and unweighted least squares. CFA indices are robust estimates from WLSMV with theta parameterization. The initial-to-final reduction resulted in a decrease of only 1.6 percentage points in explained variance, while improving residual and incremental fit.

### 3.4 Confirmatory factor analysis and sensitivity analyses

The one-factor ERP-IA-6 model was evaluated in the independent confirmatory subsample using WLSMV with theta parameterization. The model converged normally. The robust model test was statistically significant, χ^2^(9) = 72.03, *p* < 0.001. Robust incremental fit indices were favorable, CFI = 0.981 and TLI = 0.968, and the standardized residual was low, SRMR = 0.019. In contrast, the robust RMSEA was elevated, RMSEA = 0.118, 90% CI [0.093, 0.145].

All standardized factor loadings were positive, statistically significant, and high, ranging from 0.839 to 0.930. The proportion of variance explained in the individual indicators ranged from 0.704 to 0.866 (Table 2).

**Table 4:** CFA sensitivity analyses for the ERP-IA-6.

Model	*n*	Loading range	Robust χ² (df)	Robust CFI	Robust TLI	Robust RMSEA [90% CI]	SRMR
WLSMV excluding uniform-response cases	140	0.646– 0.837	24.04 (9)	0.975	0.958	0.097 [0.039, 0.154]	0.038
ULSMV estimator	272	0.832– 0.931	72.39 (9)	0.981	0.968	0.118 [0.093, 0.145]	0.019
WLSMV with re1–re2 residual covariance	272	0.814– 0.935	25.50 (8)	0.993	0.986	0.077 [0.049, 0.107]	0.011

The first sensitivity analysis excluded the 132 participants who selected the same response category across all six ERP-IA-6 items. The ULSMV analysis evaluated estimator sensitivity in the complete confirmatory subsample. The correlated-residual model was diagnostic and freely estimated the residual covariance between re1 and re2. The standardized residual association between re1 and re2 was .304, 95% CI [.220, .388], *p* < .001. The correlated-residual model was not retained as the primary model because the parameter was identified post hoc and requires substantive justification based on item content.

Inspection of modification indices identified one localized residual association exceeding 10, between re1 and re2 (MI = 11.20; expected standardized parameter change = 0.349). A diagnostic model in which this residual covariance was freely estimated showed improved robust fit, χ^2^(8) = 25.50, *p* = 0.001, CFI = 0.993, TLI = 0.986, RMSEA = 0.077, 90% CI [0.049, 0.107], and SRMR = 0.011. The standardized residual correlation between re1 and re2 was 0.304, 95% CI [0.220, 0.388], *p* < 0.001. Because this parameter was identified through a *post hoc* diagnostic and requires substantive justification based on item content, the model without correlated residuals was retained as the primary model, whereas the correlated-residual model was treated as a sensitivity analysis.

Estimator sensitivity was also examined using ULSMV. The resulting estimates were virtually identical to those obtained with WLSMV: robust CFI = 0.981, TLI = 0.968, RMSEA = 0.118, 90% CI [0.093, 0.145], and SRMR = 0.019, with standardized loadings ranging from 0.832 to 0.931. Thus, the elevated RMSEA was not attributable primarily to the selection of WLSMV rather than ULSMV.

Response-pattern sensitivity was evaluated because 132 participants in the confirmatory subsample (48.5%) selected the same response category across all six retained items. After these uniform-response cases were excluded, the model continued to converge in the remaining 140 participants. Standardized factor loadings ranged from 0.646 to 0.837, and the model retained favorable incremental and residual fit, robust CFI = 0.975, TLI = 0.958, and SRMR = 0.038. The robust RMSEA decreased to 0.097, 90% CI [0.039, 0.154], but remained above conventional guidelines. These results indicate that uniform responding contributed to the magnitude of the associations among the items but did not fully account for the one-factor structure (Table 4).

### 3.5 Reliability, AVE-based convergent evidence, and shared-method diagnostics

In the total sample, Cronbach’s alpha was 0.935, standardized alpha was 0.935, ordinal alpha was 0.945, and model-based omega was 0.948. Composite reliability estimated from the confirmatory model was 0.959, and the average variance extracted was 0.794. These results indicated high score consistency and substantial common variance among the six indicators (Table 5).

**Table 5:** Reliability, AVE-based convergent evidence, and shared-method diagnostics for the ERP-IA-6.

Indicator	Analytic sample	Estimate
Reliability and AVE-based convergent evidence		
Cronbach’s alpha	Total, *N* = 538	0.935
Standardized alpha	Total, *N* = 538	0.935
Ordinal alpha	Total, *N* = 538	0.945
Model-based omega	Total, *N* = 538	0.948
Composite reliability	CFA, *n* = 272	0.959
Average variance extracted	CFA, *n* = 272	0.794
Average interitem correlation	Total, *N* = 538	0.705
Shared-method diagnostics		
Variance explained by the dominant factor	Total, *N* = 538	74.0%
Loading range in the single-factor diagnostic	Total, *N* = 538	0.834–0.896
Uniform responses across the six items	CFA, *n* = 272	132 (48.5%)

Composite reliability and average variance extracted were calculated from the unrounded standardized CFA parameters. The single-factor diagnostic was not interpreted as a definitive test of common-method bias because the empirical analyses supported a dominant unidimensional structure. The influence of uniform response patterns was further examined through sensitivity analyses reported in Table 4.

A polychoric single-factor diagnostic conducted in the total sample showed that the first factor accounted for 74.0% of the standardized item variance, with loadings ranging from 0.834 to 0.896. Although the initial conceptual framework considered theoretically distinguishable regulatory processes, the exploratory and confirmatory analyses supported a dominant unidimensional structure in the present sample. Consequently, the single-factor diagnostic cannot determine whether the high proportion of explained variance reflects the substantive integration of these regulatory processes, shared-method variance, or a combination of both, and it was therefore not interpreted as a definitive test of common-method bias. Instead, this result was considered alongside the high average interitem correlation and the response-pattern sensitivity analyses. After uniform responders were excluded, the items retained acceptable factor loadings and the model continued to show favorable CFI, TLI, and SRMR values, indicating that the observed factor structure was not solely attributable to uniform response behavior. Nevertheless, the high item homogeneity suggests that shared wording, the common response format, and other method-related influences cannot be ruled out.

### 3.6 Measurement invariance across sex

Measurement invariance was evaluated in the confirmatory subsample across women (n = 142) and men (*n* = 130). All five response categories were represented for every item in both groups, and the configural, threshold, metric, and scalar models converged normally.

The configural model yielded robust CFI = 0.964, TLI = 0.939, and RMSEA = 0.165. Imposing equality constraints on item thresholds produced CFI = 0.974, TLI = 0.974, and RMSEA = 0.202. The metric model, which constrained thresholds and factor loadings, yielded CFI = 0.971, TLI = 0.975, and RMSEA = 0.170. The scalar model, which additionally constrained intercepts, produced CFI = 0.973, TLI = 0.980, and RMSEA = 0.170 (Table 6).

**Table 6:** Ordinal measurement invariance of the ERP-IA-6 across sex.

Model	Robust χ^2^	*df*	Robust CFI	Robust TLI	Robust RMSEA	SRMR	ΔCFI	ΔRMSEA	ΔSRMR	Robust Δχ^2^(*df*)	*p*
Configural	92.70	18	0.964	0.939	0.165	0.0221	—	—	—	—	—
Threshold	80.70	30	0.974	0.974	0.202	0.0221	0.0106	0.0370	0.0000	16.588 (12)	0.1658
Metric	88.00	35	0.971	0.975	0.170	0.0223	−0.0032	−0.0322	0.0002	8.570 (5)	0.1275
Scalar	88.70	40	0.973	0.980	0.170	0.0223	0.0021	0.0000	0.0000	2.100 (5)	0.8351

Women: *n* = 142; men: *n* = 130. The threshold model constrained item thresholds; the metric model constrained thresholds and factor loadings; and the scalar model additionally constrained item intercepts. Changes refer to the immediately preceding model. Positive changes in CFI and negative changes in RMSEA indicate improved rather than deteriorated fit. All robust scaled chi-square difference tests were nonsignificant. Although robust RMSEA remained elevated and behaved nonmonotonically, CFI and SRMR remained stable; therefore, measurement-invariance evidence was interpreted as favorable but cautious.

Robust scaled chi-square difference tests were nonsignificant for the threshold versus configural comparison, Δχ^2^(12) = 16.59, *p* = 0.166; the metric versus threshold comparison, Δχ^2^(5) = 8.57, *p* = 0.128; and the scalar versus metric comparison, Δχ^2^(5) = 2.10, *p* = 0.835. Changes in robust CFI across successive models were 0.0106, −0.0032, and 0.0021, respectively, whereas changes in SRMR were 0.0000, 0.0002, and 0.0000. Thus, the equality constraints did not produce a meaningful deterioration in incremental or residual fit.

Overall, the results provided favorable evidence of threshold, metric, and scalar invariance across sex. However, robust RMSEA values remained elevated and behaved nonmonotonically across the multigroup sequence. Consequently, the invariance evidence should be interpreted as favorable but cautious and should be based primarily on the nonsignificant robust difference tests and the stability of CFI and SRMR rather than on RMSEA alone.

## 4 Discussion

The present study aimed to develop and examine the initial psychometric properties of the AI-Assisted Psychoemotional Regulation Scale (ERP-IA-6) among Peruvian adults. Overall, the results provide favorable initial evidence for a brief, unidimensional, and psychometrically consistent measure designed to assess the perceived use of artificial intelligence tools as support in everyday psychoemotional regulation processes. This contribution is relevant because recent literature has rapidly advanced in the study of chatbots, conversational agents, and generative systems oriented toward emotional well-being, yet few brief instruments specifically assess the perceived regulatory function of AI while differentiating it from dependence, attachment, trust, or problematic use.

Before empirical evaluation, the initial eight-item pool was reviewed by five psychologists with expertise relevant to psychological assessment and the intended construct, and all items obtained Aiken’s V coefficients above 0.80. Preliminary empirical analyses subsequently showed that the six retained items displayed sufficient variability, used the full range of response categories, and did not present problematic floor or ceiling effects. Corrected item–total correlations ranged from 0.780 to 0.843, and deleting any item did not increase the internal consistency of the scale. These results support the discrimination and relevance of the six retained indicators. Nevertheless, their high correlations should be interpreted cautiously because they may reflect both substantive internal cohesion and close semantic proximity among the processes assessed.

Evidence of internal structure supported a one-factor solution. In the exploratory subsample, parallel analysis suggested retaining a single factor. The final six-item exploratory solution showed factor loadings ranging from 0.750 to 0.872 and explained 67.4% of the standardized item variance. In the independent confirmatory subsample, all standardized loadings remained high, ranging from 0.839 to 0.930, and the factor explained between 70.4 and 86.6% of the variance in the individual indicators. These results suggest that participants did not clearly differentiate AI-assisted regulation into independent subcomponents but instead perceived it as an integrated experience involving emotional identification, cognitive organization, interpretation of affective experience, reappraisal, and modulation of emotional responses.

This result can be interpreted from the perspective of process models of emotion regulation. The tradition initiated by Gross (2002) and later developed through differentiated models of strategies, stages, and regulatory orientation allows emotion regulation to be understood as a process involving monitoring, evaluation, selection, and implementation of responses (Goldenberg et al., 2016; Martínez-Priego et al., 2024). From this perspective, the ERP-IA-6 does not measure general emotion regulation or classical intrapsychic strategies, but rather the perceived support that an external technological resource provides during regulatory activity. The observed unidimensionality is compatible with the possibility that users perceive AI as offering a global scaffolding function rather than a sequence of clearly separable strategies (Growney and English, 2025; Martínez et al., 2026). However, this unidimensional structure also means that the scale does not distinguish which specific regulatory strategy—such as emotional labeling, cognitive reappraisal, response modulation, or monitoring—is being supported in each individual case.

The final six-item version was more parsimonious than the initial eight-item version. Item refinement was conducted exclusively in the exploratory subsample, whereas the retained structure was subsequently examined in an independent confirmatory subsample. The two items were not eliminated because of inadequate factor loadings. Instead, they were removed because their content overlapped with regulatory functions already represented by the retained indicators: one referred to reflection before reacting, and the other described general support for regulating emotions. The initial eight-item version explained 69.0% of the standardized variance, whereas the six-item version explained 67.4%, representing a reduction of only 1.6 percentage points. At the same time, the six-item exploratory solution showed improved RMSR, TLI, and RMSEA values. Thus, the reduction primarily reflected a balance between conceptual coverage, brevity, and avoidance of excessive redundancy rather than an attempt to maximize reliability or remove statistically weak items (Wongvorachan and Bulut, 2025).

The confirmatory results require a differentiated interpretation. The primary one-factor model showed favorable robust incremental indices, CFI = 0.981 and TLI = 0.968, and a low standardized residual, SRMR = 0.019. However, the robust RMSEA remained elevated, RMSEA = 0.118, 90% CI [0.093, 0.145]. Therefore, the confirmatory model should not be characterized as showing uniformly adequate global fit. Rather, the findings support a strong factor-loading pattern and favorable incremental and residual fit, together with a limitation in approximate absolute fit.

The elevated RMSEA cannot be attributed exclusively to the small number of degrees of freedom. Although RMSEA can falsely indicate poor fit in models with few degrees of freedom, its magnitude may also be influenced by model size, factor loadings, the treatment of ordinal indicators, estimator choice, and localized model misspecification (Kenny et al., 2015; Shi and Maydeu-Olivares, 2020). In the present study, a sensitivity analysis using ULSMV produced results that were virtually identical to those obtained with WLSMV, including a robust RMSEA of 0.118. Consequently, the unfavorable RMSEA was not explained primarily by the choice between these two ordinal estimators.

Residual diagnostics identified one localized association between re1 and re2, with a modification index of 11.20. A diagnostic model that freely estimated this residual covariance produced substantially improved fit, with CFI = 0.993, TLI = 0.986, RMSEA = 0.077, and SRMR = 0.011. The standardized residual association was 0.304. This result indicates that part of the global misfit was concentrated in a specific pair of items rather than distributed uniformly across the scale. Nevertheless, because the residual covariance was identified *post hoc* and requires a clear substantive explanation based on item wording, the model without correlated residuals was retained as the primary representation. The modified model should therefore be interpreted as a diagnostic sensitivity analysis rather than as definitive evidence supporting a more complex final structure.

A further sensitivity analysis examined the influence of uniform response patterns. Uniform responses across the six retained items were observed in 48.5% of the confirmatory subsample. When these cases were temporarily excluded, factor loadings remained acceptable, ranging from 0.646 to 0.837, and the model retained favorable CFI (0.975), TLI (0.958), and SRMR (0.038). The RMSEA decreased from 0.118 to 0.097 but remained elevated. These results suggest that uniform responding strengthened the magnitude of the interitem associations but did not fully account for the unidimensional structure. Accordingly, such patterns were retained in the primary analyses because identical responses across a brief homogeneous scale do not, by themselves, demonstrate careless or invalid participation.

The internal consistency of the ERP-IA-6 was high. Cronbach’s alpha was 0.935, ordinal alpha was 0.945, and model-based omega was 0.948. Composite reliability was 0.959, and the average variance extracted was 0.794. These results indicate that the indicators share a substantial proportion of common variance and support the interpretation of a total ERP-IA-6 score. However, the high magnitude of these coefficients should not be regarded as sufficient evidence of validity. In brief instruments, high reliability may coexist with semantic redundancy, common response tendencies, or shared method variance. Therefore, interpretation should also rely on content coverage, internal structure, sensitivity analyses, and future evidence based on relations with external variables.

Common method bias represents an additional interpretive concern because all indicators were positively worded self-report items administered in the same questionnaire at a single time point. A single-factor polychoric diagnostic indicated that the first factor accounted for 74.0% of the standardized item variance, with loadings ranging from 0.834 to 0.896. However, because the empirical analyses supported a dominant one-factor structure, this diagnostic could not distinguish substantive integration of the regulatory processes from shared-method variance. It should therefore not be interpreted as proving either the presence or absence of common method bias. The high average interitem correlation (0.705), the prevalence of uniform responses, and the high reliability estimates suggest that method-related influences cannot be ruled out, even though the factor structure remained interpretable after uniform responders were excluded.

Measurement invariance across sex provided favorable but cautious evidence of score comparability. The confirmatory subsample included 142 women and 130 men, and all five response categories were represented for every item in both groups. The configural, threshold, metric, and scalar models converged normally. Robust scaled chi-square difference tests were nonsignificant for the threshold versus configural model, the metric versus threshold model, and the scalar versus metric model. Changes in CFI and SRMR were also small and did not indicate meaningful deterioration after equality constraints were imposed. These findings support threshold, metric, and scalar invariance across women and men.

Nevertheless, RMSEA values remained elevated and behaved nonmonotonically across the invariance sequence. The robust RMSEA values were 0.165 for the configural model, 0.202 for the threshold model, and 0.170 for both the metric and scalar models. Therefore, the invariance conclusion should be based primarily on the nonsignificant robust difference tests and the stability of CFI and SRMR rather than on the RMSEA. The results provide initial support for cautious comparisons of ERP-IA-6 scores across sex, but replication with larger group-specific samples remains necessary. This consideration is consistent with previous studies demonstrating the importance of evaluating sex and cultural invariance before interpreting group differences in brief emotion regulation instruments (Burghart et al., 2023; Zhao et al., 2024).

The findings also dialogue with recent literature on AI, chatbots, and emotional well-being. Reviews of mental health chatbots show rapid growth in conversational tools based on generative models, although methodological heterogeneity, differences in evaluation quality, and cautions regarding clinical use remain substantial (Hua et al., 2025; Nyakhar and Wang, 2025). In this regard, the ERP-IA-6 does not assess therapeutic efficacy or objective symptom reduction, but rather a subjective perception of regulatory support. This distinction is central: a high score indicates that an individual perceives AI as helping to organize, understand, reinterpret, or modulate an emotional experience, but it does not imply that the interaction is clinically effective, safe, or equivalent to a validated psychological intervention.

The scale also differs from instruments oriented toward AI dependence, attachment, emotional trust, or problematic use. Whereas measures of conversational AI dependence, problematic ChatGPT use, AI attachment, or emotional dependence focus on relational bonds, maladaptive patterns, motivations, or psychological risks, the ERP-IA-6 is located in a different conceptual space: the perceived functional use of AI as a psychoemotional regulation resource. This distinction avoids automatically pathologizing emotionally oriented AI use and enables examination of an emerging phenomenon—the use of generative systems as cognitive-affective scaffolds—without assuming dependence, relational substitution, or psychological impairment.

From a theoretical perspective, the findings are compatible with the proposition that AI may operate as a form of external scaffolding for emotion regulation. Recent proposals involving affective agents, extrinsic regulation, and multimodal systems suggest that intelligent technologies may participate in emotional labeling, cognitive reappraisal, generation of alternative interpretations, and conversational support (Pico et al., 2024; Vafafar, 2025; Yu et al., 2025). Nevertheless, the present study does not demonstrate that AI objectively improves emotion regulation. It shows only that users perceive AI as providing regulatory support. Maintaining this distinction avoids a deterministic interpretation and separates perceived assistance from effective psychological mechanisms, behavioral change, and clinically verifiable outcomes.

The practical implications of the ERP-IA-6 are primarily evaluative and research-oriented. Its brief and unidimensional structure may facilitate its inclusion in studies of digital emotional literacy, subjective well-being, psychoeducational support, professional help-seeking, and patterns of human–AI interaction. In educational settings, it may help identify the extent to which students or adult learners perceive generative systems as resources for emotional organization and coping. In clinical or counseling research, it may serve as a descriptive complementary measure when examining technology-mediated coping, but it should not be used as a diagnostic instrument or as evidence that an AI interaction is therapeutically adequate.

The unidimensional score also has a practical limitation. Although it facilitates brief evaluation and reduces respondent burden, it does not identify which regulatory subprocesses are most involved or whether the person is using adaptive or maladaptive strategies. Two individuals may obtain similar scores while using AI for different purposes, such as emotional labeling, reassurance seeking, cognitive reappraisal, avoidance, or repetitive rumination. Future studies should therefore examine the ERP-IA-6 alongside more differentiated measures of emotion regulation and technology use.

The ethical interpretation of the construct requires particular care. Recent literature warns that AI-mediated emotional support may activate trust, pseudo-empathy, and socioemotional projection that are not equivalent to human reciprocity or authentic therapeutic presence (Saracini et al., 2025; Volpato et al., 2025). Similarly, although generative systems may simulate listening, validation, or mentalization, they lack clinical intentionality, moral responsibility, and relational commitment in the human sense (Yirmiya and Fonagy, 2025). For this reason, the ERP-IA-6 should be understood as a measure of perceived regulatory support, not as an indicator of therapeutic alliance, clinical quality, safety, or suitability of the interaction.

This study has several limitations. First, the cross-sectional design prevents conclusions regarding directionality or causality. It cannot be determined whether people with greater regulatory needs turn more frequently to AI, whether AI use increases perceived regulation, or whether both depend on other individual or contextual factors. Second, convenience sampling limits population representativeness. Although participants ranged from 18 to 81 years, 81.6% were between 18 and 29 years of age, and the sample was predominantly university educated and composed of AI users. The findings should therefore be generalized to the broader Peruvian adult population with caution.

The wide age range should also not be interpreted as evidence that the scale functions equivalently throughout adulthood. Older adults were sparsely represented, and measurement invariance across age groups was not examined. Consequently, the results describe a predominantly younger adult sample with some age heterogeneity rather than a sample capable of supporting conclusions about older adulthood. Future research should recruit sufficiently large and balanced age groups to test whether item thresholds, factor loadings, and score interpretations remain comparable across the adult lifespan.

Third, the scale was based exclusively on self-report and may therefore be influenced by favorable attitudes toward AI, recall limitations, response styles, social desirability, and shared questionnaire context. The high first-factor variance, elevated interitem correlations, and prevalence of uniform responses indicate that common method influences cannot be excluded. Fourth, although the findings supported a consistent unidimensional pattern, the elevated RMSEA in the primary CFA and multigroup models indicates that absolute model fit should continue to be evaluated in independent samples. The localized residual association between re1 and re2 also warrants further examination of item wording and potential content overlap.

Fifth, the study did not examine temporal stability or external validity using criteria such as general emotion regulation, psychological distress, subjective well-being, loneliness, perceived social support, AI dependence, attachment to AI, or professional help-seeking. Although AVE provided favorable evidence that the items converged on the intended latent factor, it does not establish convergent, discriminant, predictive, or criterion validity in relation to external constructs. The ERP-IA-6 should therefore be considered an instrument with favorable initial internal evidence rather than a fully validated measure for all research or applied contexts.

Future studies should examine test–retest reliability, predictive validity, convergent and discriminant relations, and performance across different age, educational, technological, national, and cultural groups. Replication should also evaluate the scale using larger independent samples, longitudinal designs, and complementary psychometric approaches, including item response theory or network analyses when theoretically appropriate. Cognitive interviewing could further clarify how participants interpret the distinction between AI-assisted regulation, reassurance seeking, emotional dependence, and relational substitution.

It will also be important to determine whether AI-assisted regulation functions as a protective resource, a compensatory response, or an indicator of vulnerability depending on the user profile, emotional context, and type of interaction with the system. The ERP-IA-6 opens a line of research that should advance with methodological rigor and ethical caution, avoiding both the idealization of AI and its automatic pathologization. Taken together, the findings support the ERP-IA-6 as a brief and psychometrically promising measure of perceived AI-assisted psychoemotional regulation among Peruvian adults, while emphasizing the need for replication, external validation, and cautious interpretation of its scores.

## 5 Conclusion

In conclusion, the ERP-IA-6 showed favorable initial evidence of content validity, unidimensional internal structure, score reliability, convergent validity, and measurement invariance across sex among Peruvian adults. The findings support its use as a brief measure of the perceived use of artificial intelligence as support in everyday psychoemotional regulation processes, including the identification, organization, understanding, reinterpretation, and modulation of emotional experiences. Nevertheless, the elevated RMSEA values, localized residual dependence, high item homogeneity, convenience sampling, and absence of external criterion measures require cautious interpretation. ERP-IA-6 scores should be understood as indicators of perceived regulatory support rather than evidence of effective emotion regulation, clinical benefit, therapeutic safety, or equivalence to professional psychological care. The scale therefore represents a parsimonious and psychometrically promising instrument for investigating an emerging phenomenon in human–AI interaction, while requiring replication and further evidence of temporal stability, external validity, and measurement equivalence across age, cultural, and technological-use groups.

## Data Availability

The original contributions presented in the study are included in the article/Supplementary material, further inquiries can be directed to the corresponding author.
