# Supplementary material for: Development and validation of the AI-assisted psychoemotional regulation scale (ERP-IA-6)
Source: Front Psychol. 2026 Sep 9;17:1917798. doi: 10.3389/fpsyg.2026.1917798 (PMC13598090; doi:10.3389/fpsyg.2026.1917798)
Supplement: Supplementary file 1 [file Supplementary_file_1.docx]

Supplementary Material

# 1 Instrumento

**Escala Breve de Regulación Psicoemocional Asistida por IA (ERP-IA-6)**

La Escala Breve de Regulación Psicoemocional Asistida por IA (ERP-IA-6) está compuesta por seis ítems positivos que evalúan el uso percibido de herramientas de inteligencia artificial como apoyo en procesos cotidianos de identificación, organización, comprensión, reinterpretación y modulación de experiencias emocionales. Los ítems se responden mediante una escala tipo Likert de cinco puntos, desde 1 = totalmente en desacuerdo hasta 5 = totalmente de acuerdo. Una mayor puntuación indica una mayor percepción de uso de la inteligencia artificial como recurso de apoyo para la regulación psicoemocional.

| **Código** | **Ítem** |
| --- | --- |
| RE1 | Cuando me siento emocionalmente alterado/a, la IA me ayuda a identificar mejor lo que estoy sintiendo. |
| RE2 | La IA me ayuda a ordenar mis pensamientos cuando experimento malestar emocional. |
| RE3 | Consultar con IA me permite comprender con mayor claridad lo que me ocurre emocionalmente. |
| RE4 | La interacción con IA me ayuda a interpretar una situación difícil de una manera más serena. |
| RE5 | Después de usar IA, suelo comprender mejor por qué reaccioné de cierta manera. |
| RE6 | Cuando experimento estrés o ansiedad, recurro a la IA para recuperar la calma. |

**Opciones de respuesta**

| **Valor** | **Opción de respuesta** |
| --- | --- |
| 1 | Totalmente en desacuerdo |
| 2 | En desacuerdo |
| 3 | Ni de acuerdo ni en desacuerdo |
| 4 | De acuerdo |
| 5 | Totalmente de acuerdo |

**Interpretación del puntaje**

El puntaje total se obtiene sumando o promediando las respuestas de los seis ítems. Puntuaciones más altas indican una mayor percepción de que la inteligencia artificial funciona como apoyo para identificar, organizar, comprender, reinterpretar y calmar experiencias emocionales cotidianas. La escala no debe interpretarse como una medida de eficacia clínica, diagnóstico psicológico, dependencia de IA o sustitución de apoyo profesional.
